# Editorial: Robot-assisted rehabilitation for neurological disorders

**DOI:** 10.3389/frobt.2022.1014681

**Published:** 2022-09-15

**Authors:** Giuseppe Carbone, Rogério Sales Gonçalves

**Affiliations:** ^1^ DIMEG, University of Calabria, Cosenza, Italy; ^2^ School of Mechanical Engineering, Federal University of Uberlandia, Uberlandia, Brazil

**Keywords:** robotics in clinical practice, exoskeleton, wearable devices, rehabilitation, and assistive robotics

## Introduction

The mortality rate of people with neurological disorders, such as stroke, Parkinson’s disease, spinal cord injury, cerebral palsy, multiple sclerosis is decreasing due to advancements in medical research, as mentioned for example in (Gonçalves et al., 2012; Gonçalves et al., 2016). However, neurological disorders can result in long-term disabilities and significant improvements are still required for cost and time-effective recovery of neurological functions such as discussed in (Goncalves et al., 2017). In recent years, research on rehabilitation robotics has progressed from proposing solutions for the clinical field to more portable solutions tailored to the user’s requirements. However, more efforts are still needed in the development of robotic solutions for neurological rehabilitation that are accessible to a large number of patients as outlined in multiple works such as, for example, reported in (Volpe et al., 2009; Loureiro et al., 2011; Yamamoto et al., 2014; Copilusi et al., 2015; Gherman et al., 2019; Gonçalves et al., 2019; Vaida et al., 2020).

This Research Topic aims at presenting innovative robotic solutions to boost the rehabilitation process for neurological disorders. This Issue highlight recent advances in the development of these robotic devices including a comprehensive review of recent developments in the area of rehabilitation robotics, information, and recent achievements on both therapeutic and assistive robots, focuses on the state-of-the-art and representative advancements in the design, simulation, control, analysis, implementation, serious games, and validation of rehabilitation robotic systems.

This Research Topic contains feature articles as focused on theoretical and experimental results dealing with techniques for the design, simulation, and control systems for rehabilitation devices for upper and lower limbs including serious games and virtual reality. The presented solutions explore the development of devices that are cost-effective with high impact and potential for future developments to clinical applications. Still the Research Topic will require careful research attention in the near future for progressing human wealth.

## Advances in rehabilitation devices for neurological disorders

In a first article, (Alves et al.), the authors outline the development of serious games strategies used together with a cable-driven robot applied for bimanual rehabilitation. Cable-driven robots can be an ideal fit for performing post-stroke rehabilitation due to their specific features in function have small and lightweight moving parts and a relatively large workspace. The results demonstrate the engineering feasibility and effectiveness of the proposed cable-driven robot as a valuable tool to augment the conventional physiotherapy protocols and for providing reliable measurements of the patient’s rehabilitation performance and progress. The second article (Dragusanu et al.) presents a design and prototype of an underactuated hand exoskeleton with fingers coupled by a gear-based differential. Exoskeletons devices represent a promising opportunity for rehabilitation and assistance to people presenting neurological diseases. However, there are still some limits to the diffusion of robotic technologies for neurorehabilitation, notwithstanding their technological developments and evidence of clinical effectiveness. In the article (Mo et al.), the authors present an anti-disturbance sliding mode control for a novel variable stiffness actuator to be applied for lower limb rehabilitation. Lower limb exoskeletons are widely used for rehabilitation training of patients suffering from neurological disorders. In this paper, a novel load-adaptive variable stiffness actuator is used to design an ankle exoskeleton with a sliding mode controller based on a disturbance observer. The simulation and experimental verification are performed, and the wearing experiment confirms that the controller can realize zero-impedance control of the designed ankle exoskeleton. The design of a suspension lever mechanism with elastic elements for a robotic rehabilitation system for the lower limbs was presented in the fourth article, (Rybak et al.). The article discusses the design of a suspended lever mechanism with elastic elements, which is used as a safety device in a robotic system for the rehabilitation of the lower limbs. The article analyzes the existing mechanical structures of devices for rehabilitation, identifies the problems of operation, design, and safety systems, and suggests a modern design for the device. The article addresses the issues present in the current mechanical designs with a brief discussion on the system architecture. The author of the last article (Liu et al.) presented a systematic review and meta-analysis of the effect of virtual reality on balance function in children with cerebral palsy. Virtual reality therapy is popular in treating children with cerebral palsy as a modern technology for rehabilitation. The systematic review and meta-analysis included all randomized controlled trials that included children with cerebral palsy. Results showed that the virtual reality intervention was beneficial for balance, however, cautious implementation is needed in clinical applications.
